# Serum Amyloid A Is Present in Human Saccular Intracranial Aneurysm Walls and Associates With Aneurysm Rupture

**DOI:** 10.1093/jnen/nlab086

**Published:** 2021-09-17

**Authors:** Nora Huuska, Eliisa Netti, Riikka Tulamo, Satu Lehti, Behnam Rezai Jahromi, Petri T Kovanen, Mika Niemelä

**Affiliations:** 1 From the Doctoral Programme in Biomedicine, Doctoral School in Health Sciences, University of Helsinki, Helsinki, Finland; 2 Neurosurgery Research Group, Biomedicum, Helsinki, Finland; 3 Department of Neurosurgery, Helsinki University Hospital and University of Helsinki, Helsinki, Finland; 4 Department of Vascular Surgery, Helsinki University Hospital and University of Helsinki, Helsinki, Finland; 5 Wihuri Research Institute, Biomedicum, Helsinki, Finland; 6 Gerontology Research Center, Faculty of Sport and Health Sciences, University of Jyväskylä, Jyväskylä, Finland

**Keywords:** Inflammation, Saccular intracranial aneurysm, Serum amyloid A

## Abstract

Saccular intracranial aneurysm (sIA) rupture leads to a disabling subarachnoid hemorrhage. Chronic inflammation and lipid accumulation in the sIA wall contribute to wall degenerative remodeling that precedes its rupture. A better understanding of the pathobiological process is essential for improved future treatment of patients carrying sIAs. Serum amyloid A (SAA) is an acute-phase protein produced in response to acute and chronic inflammation and tissue damage. Here, we studied the presence and the potential role of SAA in 36 intraoperatively resected sIAs (16 unruptured and 20 ruptured), that had previously been studied by histology and immunohistochemistry. SAA was present in all sIAs, but the extent of immunopositivity varied greatly. SAA immunopositivity correlated with wall degeneration (p = 0.028) and rupture (p = 0.004), with numbers of CD163-positive and CD68-positive macrophages and CD3-positive T lymphocytes (all p < 0.001), and with the expression of myeloperoxidase, matrix metalloproteinase-9, prostaglandin E-2 receptor, and cyclo-oxygenase 2 in the sIA wall. Moreover, SAA positivity correlated with the accumulation of apolipoproteins A-1 and B-100. In conclusion, SAA occurs in the sIA wall and, as an inflammation-related factor, may contribute to the development of a rupture-prone sIA.

## INTRODUCTION

A saccular intracranial aneurysm (sIA) is a localized pouch-like dilation of a cerebral artery. A fraction of the sIAs ruptures and leads to a subarachnoid hemorrhage with ensuing disability or death (1). The current treatment methods to prevent sIA rupture are invasive and are associated with treatment-related risks of 5%–7% morbidity and 1%–2% mortality (2, 3). Therefore, it is important to better understand the pathobiology of sIA. Such understanding will guide us in clinical decision-making and will also aid us when attempting to develop new diagnostic and noninvasive preventive treatment methods for patients with a developing sIA.

The sIA walls are characterized by degenerative remodeling, including degeneration of the extracellular matrix (ECM), loss of smooth muscle cells (SMCs), chronic inflammation, and atherosclerotic changes (4–12). Conclusive pathophysiological mechanisms underlying sIA formation and rupture remain, however, unknown.

Serum amyloid A (SAA) is an acute-phase (AP) protein produced mainly in the liver in response to systemic infection and tissue damage (13). In the circulation, SAA is carried in high-density lipoprotein (HDL) particles. (14). We have previously demonstrated that sIA walls show accumulation of apolipoproteins A-1 (apoA-1) and B-100 (apoB-100), which are components of the circulating HDL and low-density lipoproteins (LDLs), respectively (15). In the sIA wall, the presence of both apoA-1 or apoB-100 were found to associate with inflammation and degenerative changes, the apoA-1-containing lipoproteins being considered to possess anti-inflammatory and the apoB-100-containing lipoproteins proinflammatory properties (16).

During acute inflammation, the plasma levels of SAA are known to increase to levels that are up to 1000-fold higher than normal. A modest but persistent increase in plasma level of SAA is detected in conditions associated with systemic chronic low-degree inflammation, among them atherosclerotic cardiovascular diseases, notably coronary artery disease (17). Importantly, SAA has been detected in atherosclerotic lesions of human coronary and carotid arteries (18). Studies in patients with atherosclerotic lesions and experiments using human cell cultures have shown that SAA induces the synthesis of various cytokines and attracts leukocytes, such as neutrophils, monocytes, and mast cells to the site of inflammation (19–22). Notably, all of these cell types have also been detected in human degenerated sIA walls (6). SAA has been also shown to play a role in the formation of abdominal aortic aneurysm in an experimental mouse model by triggering matrix metalloproteinase 2 (MMP-2) activity and ensuing elastin degradation (23). Furthermore, when added to cultured human monocytes or macrophages, SAA upregulates the expression of the ECM-degrading MMP-2 and MMP-9, which have also been detected in the human sIA walls (10, 24, 25).

In this study, we searched for the presence of SAA in the human sIA wall in an attempt to delineate its potential role in the pathobiology of this degenerative disease.

## MATERIALS AND METHODS

### Samples of Saccular Intracranial Aneurysms

A total of 36 saccular intracranial aneurysm (sIA) samples (16 unruptured and 20 ruptured) included in a previously published sIA series were studied (8–10, 26, 27). The sIA samples were resected after surgical clipping at the Department of Neurosurgery, Helsinki University Hospital (HUH), Helsinki, Finland. After harvesting, the samples were immediately snap-frozen in liquid nitrogen and stored at −80°C. For histological and immunohistochemical staining, the frozen samples were embedded in Tissue-Tek (Sakura, Alphen aan den Rijn, The Netherlands) and cryosectioned at 4 µm. Clinical data were collected from the patients’ medical records and sIA dimensions were obtained from preoperative computed tomography angiography images. The HUH Ethics Committee approved this study.

### Basic Characteristics of the Aneurysm Walls

**FIGURE 1.**
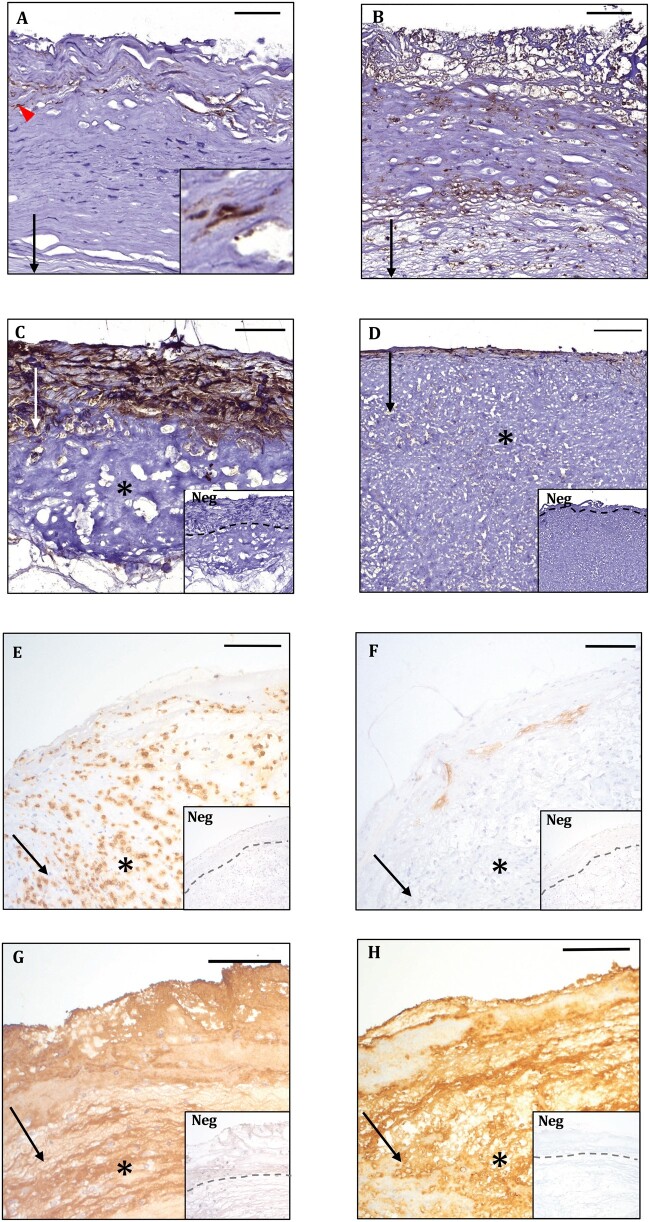
Representative images of saccular intracranial aneurysm wall types A–D (4) **(A–D**, respectively**)** presenting immunohistochemical staining for serum amyloid A scores 1 **(A)**, 2 **(B)**, and 3 **(C)**. **(E–H)** Immunohistochemical stainings of the same ruptured sIA as shown in **(C)** for matrix-metalloproteinase-9 **(E)**, CD34 **(F)**, apolipoprotein A-1 **(G)**, and apolipoprotein B-100 **(H)**. Arrows point down towards the lumen. Thrombus is indicated with an asterisk. Intracellular serum amyloid A is indicated with an arrowhead and shown as an inset in **(A)**. Negative controls are shown as insets. The interphases between the saccular intracranial aneurysm wall and thrombus are indicated by dashed lines in the insets. Positive staining is brown. Hematoxylin background staining. Scale bars: **(A, C)** = 50 µm; **(B, D–H)** = 100 µm.

The sIAs were classified into categories A–D according to the characteristics of their walls: type A (9/36; 25%), type B (12/36; 33%), and type C (11/36; 31%) (8). Only 2 samples representative for wall type D (2/36; 6%) were present and therefore they were excluded from further analyses. The following wall classification criteria have been originally published by Frösen et al (4): wall type A displayed a wall with intact endothelium and an organized layer of SMCs, type B displayed a thickened wall and a disorganized layer of SMCs, type C displayed a hypocellular wall with either myointimal hyperplasia or organized thrombus, and type D displayed a very thin hypocellular wall with organized thrombus. A demonstrative outlining of the different aneurysm wall types has been provided in a review article by Tulamo et al (28). Figure 1 describes the different wall types among the studied sIA sample series. This sIA series has also been analyzed earlier for inflammatory and lipid characteristics (Table) (8–10, 26).

### Histology and Immunohistochemistry

The histological sections were fixed with 4% paraformaldehyde at room temperature (RT) for 10 minutes, and sequentially incubated with the EnVision kit’s blocking reagent (Dako, Santa Clara, CA) and 3% normal horse serum (Vector, Burlingame, CA) at RT for 20 minutes. The sections were incubated with an anti-SAA antibody (Table) for 60 minutes at RT. The secondary detection with EnVision Kit was performed according to the manufacturer’s protocol. The sections were background stained with Mayer’s hematoxylin (Sigma-Aldrich, St Louis, MO), and embedded in a mounting aqueous medium (Faramount; Dako). An irrelevant mouse monoclonal antibody (IgG1 or IgG2a, depending on the subclass of primary antibody; Serotec, Oxford, UK) served as a substitute for the primary antibody in negative controls. Resected freshly frozen human tonsil tissue served as a positive control.

To study whether SAA-positive areas contain amyloid in the fibril form, the sections were stained with the Amyloid stain, Congo Red-staining kit (Sigma-Aldrich) according to the manufacturer’s instructions for Puchtler’s modification for amyloid. Amyloid Tissue-Trol-control slides (Sigma-Aldrich) prepared from human hearts were used as positive controls.

### Analysis

The immunohistochemical stainings were scanned using a digital slide scanner (3DHISTEC, Budapest, Hungary). Positive stainings for SAA and their histological locations were analyzed semiquantitatively from the sections: the samples were scored as 1–3 depending on the extent and location of the stained area (Fig. 1). SAA score 1 represented a wall with a few positively stained cells and possibly a small amount of ECM-positive area. SAA score 2 represented a wall with either scattered SAA-positive cells or a local cluster of them, accompanied by an EMC-positive area, the total positively stained area covering <50% of the sIA wall’s area. SAA score 3 represented a wall with widespread positive cells and ECM-positive area, the positive signal covering over 50% of the sIA wall’s area.

The assigned SAA score was then compared with sIA wall type, sIA rupture, lipids, lipoproteins, and inflammatory markers, as defined in our previous studies (8–10, 26) and shown in the Table. The degree of overlap of an SAA-positive area with areas positive for MMP-9, CD34, apoA-1, and apoB-100 (Fig. 1) was defined as complete, partial, or absent.

### Statistics

Data analysis was performed using the IBM SPSS Statistics Software, version 25. For categorical variables, proportions were calculated, and the Fisher exact test was used. For continuous variables, median and range were calculated, and Spearman correlation tests were used. p Values < 0.05 were considered statistically significant.

## RESULTS

### SAA Is Present in All sIA Walls and Associates With Wall Degeneration and Rupture

**FIGURE 2.**
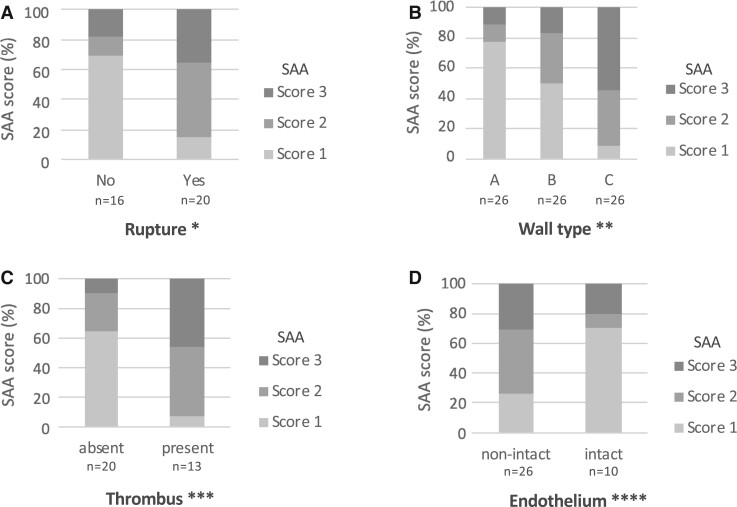
Association of serum amyloid A score with the saccular intracranial aneurysm rupture status **(A)**, wall type **(B)**, presence of intraluminal thrombus **(C)**, and presence of intact endothelium **(D)** in the saccular intracranial aneurysm wall. Fisher exact test. *p < 0.004, **p < 0.028, ***p < 0.003, ****p < 0.070.

SAA-positive staining was present in all sIA walls, and the extent and intensity of the staining varied greatly. In the sIA walls, SAA was localized both intracellularly and extracellularly typically in the adventitial layer of the wall (Fig. 1; low-magnification images in Supplementary Data Fig. S2). Of the sIA walls, 14/36 (39%) were classified as SAA score 1, 12/36 (33%) as SAA score 2, and 10/36 (28%) as SAA score 3. The greater the area of SAA-positive staining in the wall, the bigger portion of it was located extracellularly; none of the samples contained only intracellularly located SAA. The SAA-positive area overlapped partly with the CD34-positive neovessels in the adventitial side of the sIA wall. An example of a typical CD34-positive staining is presented in Figure 1F. The extent of the SAA-positive area increased in proportion with the number of CD34-positive neovessels. The staining of SAA in the sIA wall was most extensive in the ruptured sIAs, but the location of the SAA-positive area (Fig. 2), whether adventitial or luminal in the sIA wall, was independent of the aneurysm rupture. No amyloid fibrils were found in the Congo red-stainings, indicating that SAA was present in the native, rather than in fibril form in the sIA wall.

We also observed that SAA stained more extensively in the more degenerated type C than in the intact-type A sIA walls (Fig. 2), revealing that the SAA staining was associated with sIA wall degeneration. More specifically, the loss of SMCs in the sIA wall was associated with the extent of the SAA staining (p = 0.005, Fisher exact test), the loss of intact endothelium showed a positive trend with the extent of SAA staining, and the presence of fresh or organized thrombus was associated with the extent of SAA staining (Fig. 2). When these characteristics were defined either as regular or irregular, the SAA score did not associate with the size or shape of the aneurysm. Conclusively, the above findings suggest that the accumulation of SAA associates with some mechanisms leading to the degenerative remodeling of the sIA walls, as has been observed previously regarding other inflammatory markers (4–12).

### SAA in the sIA Wall Associates With Lipid Accumulation

**FIGURE 3.**
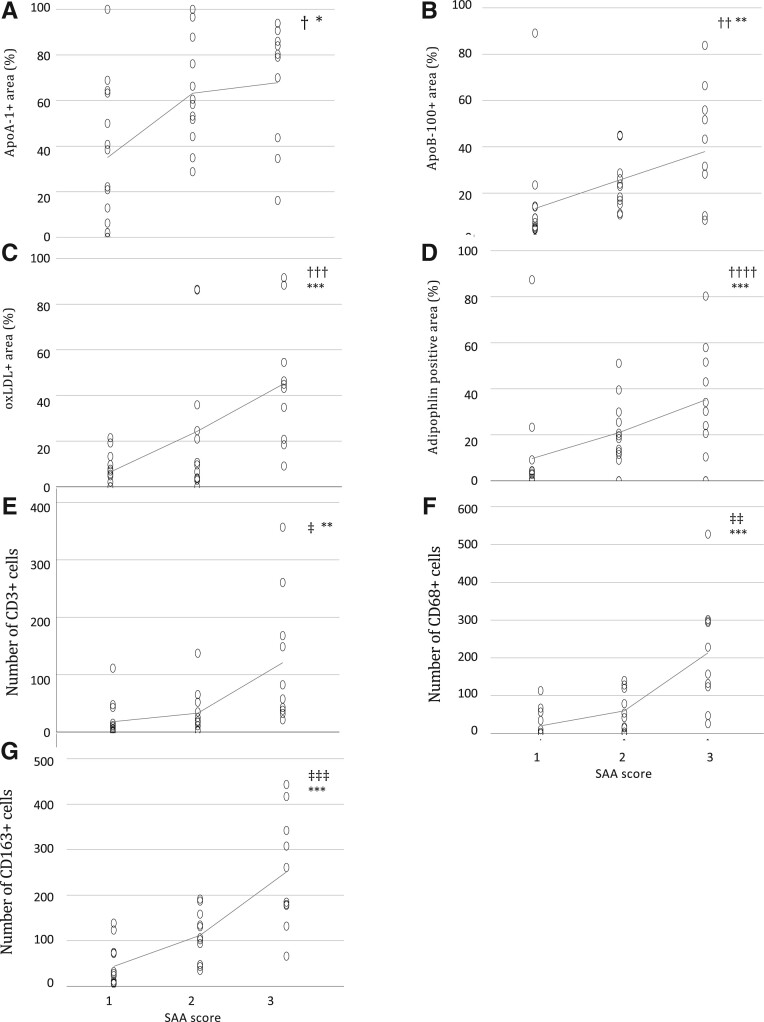
Correlations of positively stained area of apolipoprotein A-1 **(A)**, apolipoprotein B-100 (apoB-100) **(B)**, oxidized low-density lipoprotein **(C)**, and adipophilin **(D)** and number of CD3-positive **(E)**, CD68-positive **(F)**, and CD163-positive **(G)** cells with serum amyloid A scores in 36 saccular intracranial aneurysm walls. Spearman’s correlation test. *p = 0.006, **p = 0.001, ***p < 0.001, †R = 0.452, ††R = 0.526, †††R = 0.635, ††††R = 0.585, ‡R = 0.624, ‡‡R = 0.732, ‡‡‡R = 0.756.

The extent of SAA staining associated with the apoA-1-positive areas of the sIA walls (Fig. 3), and their stainings also partially overlapped, thereby revealing the presence of HDL in these areas. Moreover, the extent of SAA-positive area was associated (Fig. 3) and partly overlapped with the apoB-100-positive areas, which revealed the presence of apoB-100-containg lipoproteins that is, LDL, very LDLs, and/or intermediate-density lipoproteins in these SAA-containing areas. In most cases, the apoA-1- and apoB-100-positive stainings covered almost the entire sIA wall, whereas the SAA-positive area was limited to the adventitial side of the sIA wall; therefore, leading to only a partial overlap between lipoprotein-containing and SAA-containing areas. Examples of typical apoA-1 and apoB-100 positive stainings are shown in Figure 1G, H. In addition, SAA was associated with oxidized lipids (hydroxynonenal-positive area), and also with adipophilin (Fig. 3), a marker of intracellular lipid accumulation. However, the presence of SAA did not associate with the accumulation of neutral lipids that is, Oil Red O-positive areas in the sIA-walls (p = 0.260, R = 0.193, Spearman).

### SAA in the sIA Wall Associates With Inflammatory Markers and MMPs

**FIGURE 4.**
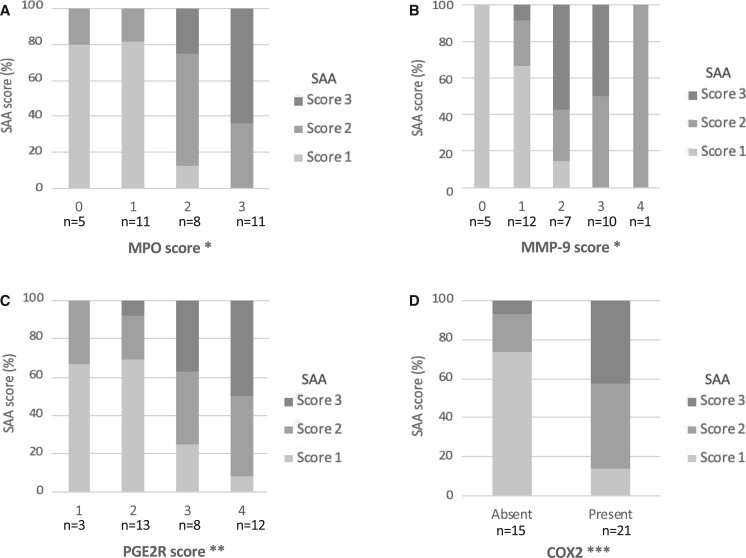
Percentual distribution of serum amyloid A scores 1–3 among the myeloperoxidase scores 0–3 **(A)**, matrix metalloprotein 9 scores 0–4 **(B)**, prostaglandin E 2 receptor scores 1–4 **(C)**, and absence/presence of cyclo-oxygenase 2 **(D)** in the 36 saccular intracranial aneurysm walls. Fisher exact test. *p < 0.001, **p = 0.027, *** p = 0.001.

The extent of SAA-positive staining in the sIA wall showed a strong positive correlation with the numbers of CD163-positive and CD68-positive macrophages, and also with those of CD3-positive T lymphocytes (Fig. 3). In the sIA walls, also mast cells were present, but SAA did not associate with the presence or density of these inflammatory cells (p = 0.127, Fisher exact test; p = 0.124, Spearman, respectively). Accumulation of SAA did associate with the expression levels of myeloperoxidase (MPO), a neutrophil-derived enzyme, and also with those of prostaglandin E2 receptors (PGE2R), and cyclo-oxygenase 2 (COX2), as indicators of inflammation (Fig. 4; representative images of the stainings in Supplementary Data Fig. S1). The MMP-9 stained mainly intracellularly, whereas MMP-2 was localized extracellularly within the ECM. In most cases, the MMP-9-positive cells were covering less than half of the wall area. SAA-positive areas in the sIA wall correlated positively (Fig. 4) and partly overlapped with MMP-9-positive areas. An example of a typical MMP-9-positive staining is presented in Figure 1E. The extent of the SAA-positive area increased in proportion with the MMP-9-positive area. In addition, all sIA walls stained positively for MMP-2, and most of them extensively.

## DISCUSSION

In this immunohistochemical study, the presence of SAA is demonstrated for the first time in the human sIA wall. Importantly, we could demonstrate that SAA associates with the degenerative remodeling and rupture of the sIA wall. SAA was found to be present in the walls of all studied 36 sIAs, but there was great variation in the extent of its presence. SAA was present both extra- and intracellularly and typically localized in the adventitial side of the wall. This study is a continuum of our earlier research of the human aneurysm disease (8–10, 26, 28), which aims to increase the basic understanding of the aneurysm pathobiology, and together with future technological advances, will hopefully aid in improving clinical applications and guidelines in the treatment of aneurysms.

The pathogenic role of SAA in human disease was first recognized, when it was discovered that SAA is the precursor of amyloid A protein in amyloid A amyloidosis. In this disease, amyloid fibrils accumulate in various organs due to misfolding of the amyloid A protein (31). Amyloid fibril deposits are also detected in cerebral amyloid angiopathy, in which β-amyloid accumulates within the cortical and leptomeningeal small blood vessel walls causing their subsequent degeneration and increasing the risk of an intracerebral hemorrhage in the elderly (32). In the sIA walls of the present immunohistochemical study, however, the Congo Red staining, which detects amyloid fibrils, was negative in all samples demonstrating that no amyloid fibrils had accumulated in the sIA walls. This finding suggests that the microenvironment in the sIA wall favors SAA occurrence rather as a soluble polypeptide than in the fibril form. However, this study was not able to reveal any specific mechanisms underlying the accumulation of SAA in the sIA walls.

There are two major potential sources for the SAA in the sIA wall: either it is derived from the circulation or synthesized locally. The circulating SAA is synthesized and secreted by the liver, and the majority of it is bound to the surface of HDL particles. Upon binding to HDL, 1 molecule of SAA displaces one or more of the several apoA-1 molecules from the HDL particle surface, upon which an AP-HDL particle is formed (33). SAA may enter the sIA wall across the luminal endothelium either as free SAA or as an HDL particle-associated molecule that is, when present in the AP-HDL (34). In our series, the SAA-positive areas partly colocalized with apoA-1-positive areas, rendering it possible that SAA, AP-HDL, and HDL coexisted in such areas. SAA molecules may enter the sIA wall from the luminal side by the centrifugal mass transport, which has been proposed to play a role for example in abdominal aortic aneurysms (35).

Within the sIA wall, free SAA and AP-HDL-associated SAA may bind via the heparin-binding sites of SAA to the negatively charged chondroitin sulfate chains of the ECM, so explaining the extracellular retention and accumulation of SAA and/or AP-HDL within the sIA wall (36), as also observed in this study. AP-HDL particles may also bind to the negatively charged biglycan molecules on macrophage surfaces, thereby blunting HDL’s anti-inflammatory effects on the macrophages (33). Moreover, some other, yet unknown factors may facilitate the binding of the SAA in the adventitial side of the aneurysm wall. In addition to the luminal entry, SAA/AP-HDL may enter the sIA wall also from the adventitial side via the microvascular sprouts derived from the adventitial *vasa vasorum*. Our observations that SAA preferably accumulates in the adventitial side of the sIA wall and that it overlaps with the localization of the CD34-positive adventitial neovessels further supports the hypothesis that circulating SAA and AP-HDL may also enter the sIA wall from its adventitial side via neovessels, which are also surrounded by hemosiderin deposition as a sign of their leakiness (8). In addition, the colocalization of SAA with neovessels may be due to the ability of SAA to promote angiogenesis by stimulating vascular endothelial growth factor receptor 2 expression that results in endothelial tube formation, as shown at least in vitro (37).

We also detected intracellularly localized SAA, which raises the possibilities of synthesis and/or cellular uptake of SAA by the cells present in the sIA wall. Although the circulating SAA is mainly produced by the hepatocytes in response to systemic inflammation as a part of the AP reaction, extrahepatic production of SAA has been observed in animal models (38). Moreover, cell culture experiments have demonstrated the ability of many human cell types to synthesize SAA, among them also the macrophages (18, 39). Since the sIA wall contains large amounts of macrophages (9), they may serve as a source of SAA in the sIA wall. However, this study cannot determine whether the intracellularly accumulated SAA results from uptake or synthesis of SAA by the macrophages—or both.

Like the atherosclerotic arterial walls, the sIA walls also contain modified lipids and lipid-laden foam cells. In this study, we demonstrate that SAA associates with the accumulation of apoB-100 in the sIA wall. Prior studies have shown that SAA may also associate with apoB-100 containing lipoproteins, which include both LDL and very-LDLs (40–42). In the atherosclerotic artery wall, SAA is known to activate the NLRP3 inflammasome inducing the transforming growth factor-β, which increases vascular biglycan expression and leads to increased LDL retention (43, 44). The NLRP3 inflammasomes have been shown to be expressed in human sIA walls, and even more so in ruptured aneurysms (45).

In this study, SAA was also associated with MPO, oxidized lipids, and adipophilin, but not with Oil-Red-O-positive lipids that is, neutral lipids, which mainly accumulate as cholesteryl ester droplets within macrophages. A plausible explanation for the lack of association with Oil-Red-O-positive lipids that is, cholesteryl ester-containing lipid droplets in foam cells, is the finding by Tam et al demonstrating that binding of SAA/AP-HDL to cell surface heparan sulfate generates a novel lipoprotein with exceptional cholesterol efflux activity from macrophages (34). Finally, oxidized LDL is shown to induce SAA expression in human monocytes, which could lead to the local expression of SAA in the monocyte-derived macrophages present in the sIA wall (46–48).

Furthermore, SAA is potentially involved in many stages of arterial inflammation and may also affect many processes shown to be present in sIA walls. Accordingly, SAA has been shown to increase reactive oxygen species leading to oxidative stress and endothelial dysfunction in vitro (49). SAA has also been shown to stimulate the migration of phagocytes, T cells, mast cells and monocytes and to promote their adhesion to the endothelium and their infiltration into the arterial wall (50–52). In this study, the accumulation of SAA in the sIA wall correlated positively with the number of inflammatory cells, including macrophages and T lymphocytes, and with the expression of inflammatory mediators, such as MPO, MMP-9, PGE2R, and COX2, which may reflect their mutual interactions also in the sIA wall. SAA is known to activate the NLRP3 inflammasome in macrophages, leading to the maturation of inflammatory cytokines such as interleukin-1 beta and interleukin-18, and initiating a type of inflammatory cell death called pyroptosis (53).

MMPs contribute to sIA wall remodeling by degrading various components of the ECM (24). Here, we found a clear positive association between the content of SAA and the sIA wall degeneration, and that accumulation of SAA associates with the expression of MMP-9 in the sIA walls. Importantly, in cultures of human monocytes and macrophages, SAA upregulates the expression of the ECM-degrading MMP-2 and MMP-9 (25), and it contributes to the formation of the abdominal aortic aneurysm by promoting elastin degradation via inducing MMP-2 activity (23). Taken together with the above observations, our finding of MMP-2 positivity in all of the degenerating sIA walls assigns a role for this enzyme in the generation of the sIA.

### Conclusion

This is the first demonstration of the presence of SAA in the sIA wall. Importantly, we also found that its accumulation associates with the sIA wall degeneration and rupture. In the sIA wall, the accumulation of SAA is positively correlated with the number of inflammatory cells, including macrophages and T lymphocytes, and with the expression of inflammatory mediators, such as MPO, MMP-9, PGE2R, and COX2. Moreover, the accumulation of SAA correlated with the accumulations of apoA-1- and apoB-100-containing lipoproteins, which may serve as carriers for SAA from the circulation to the sIA wall. These findings suggest that SAA may have a role in the degeneration of the sIA wall, thereby leading to a rupture-prone aneurysm*.* However, whether SAA accumulation actively induces sIA wall degeneration and rupture, or whether it rather represents only one component in the pathobiological process in the degeneration of the sIA wall, remains to be studied. Accordingly, the present immunohistochemical observations call for further studies aimed at uncovering the exact mechanisms of the contribution of the SAA molecules present in the sIA wall to the progression of the wall degeneration and ultimate rupture.

**TABLE.** Primary Antibodies Used in the Immunostainings

Antigen/Epitope*	Antibody Clone	Concentration (mg/L) or Dilution^†^	Manufacturer
SAA	MC1^‡^	1:100	Dako (Glostrup, Denmark)
COX2	CX-294^‡^	1:100	Dako
PGE2R	LS-A968**	1:100	LSBio (Seattle, WA)
CD68	EBM11^‡^	2.4	Dako
CD163	5C6-FAT^‡^	20	Novus Biologicals (Littleton, CO)
CD3	F7.2.38^‡^	1:500	Dako
CD31	JC70A^‡^	200	Dako
CD34	QBEnd/10^‡^	12	Novocastra (Newcastle upon Tyne, UK)
Mast cell tryptase	AA1^‡^	1:500	Dako
Mast cell chymase	CC1^‡^	1:500	Serotec (Oxford, UK)
αSMA	1A4^††^	71	Dako
apoA-1	1C5^‡^	0.10	Monosan (Uden, The Netherlands)
apoB-100	MB47^††^	1:10 000	(29, 30)
HNE	HNE^‡‡^	1:300	(29)
Adipophilin	AP125^‡^	1:10	R&D Systems (Minneapolis, MN)
ABCA1	NB400-105**	1:100	Novus Biologicals
MPO	A0398**	1.8	Dako
MMP-9	4H3^‡^	0.20	Novus Biologicals

* SAA, serum amyloid A; COX2, cyclo-oxygenase 2; PGE2R, prostaglandin E 2 receptor; αSMA, alpha-smooth muscle actin; ApoA-I, apolipoprotein A-1; ApoB-100, apolipoprotein B-100, HNE, hydroxynonenal; ABCA1, ATP-binding cassette transporter A1; MPO, myeloperoxidase; MMP-9, matrix metalloproteinase 9.

† Final concentration given, if available.

‡ Mouse monoclonal IgG1.

** Rabbit polyclonal.

^††^ Mouse monoclonal IgG2.

^‡‡^ Guinea pig polyclonal.

## Supplementary Material

nlab086_Supplementary_Data

## Notes

Nora Huuska and Eliisa Netti contributed equally to this work.

This work was supported by the Helsinki University Hospital EVO grant TYH2018316, Maire Taponen Foundation, the Finnish Medical Foundation, and Petri Honkanen Foundation. Wihuri Research Institute is maintained by the Jenny and Antti Wihuri Foundation.
