# Supplementary material for: Serum Amyloid A Is Present in Human Saccular Intracranial Aneurysm Walls and Associates With Aneurysm Rupture
Source: J Neuropathol Exp Neurol. 2021 Sep 17;80(10):966–74. doi: 10.1093/jnen/nlab086 (PMC9278718; doi:10.1093/jnen/nlab086)
Supplement: nlab086_Supplementary_Data [file nlab086_supplementary_data.pdf]

Supplemental Figure 1

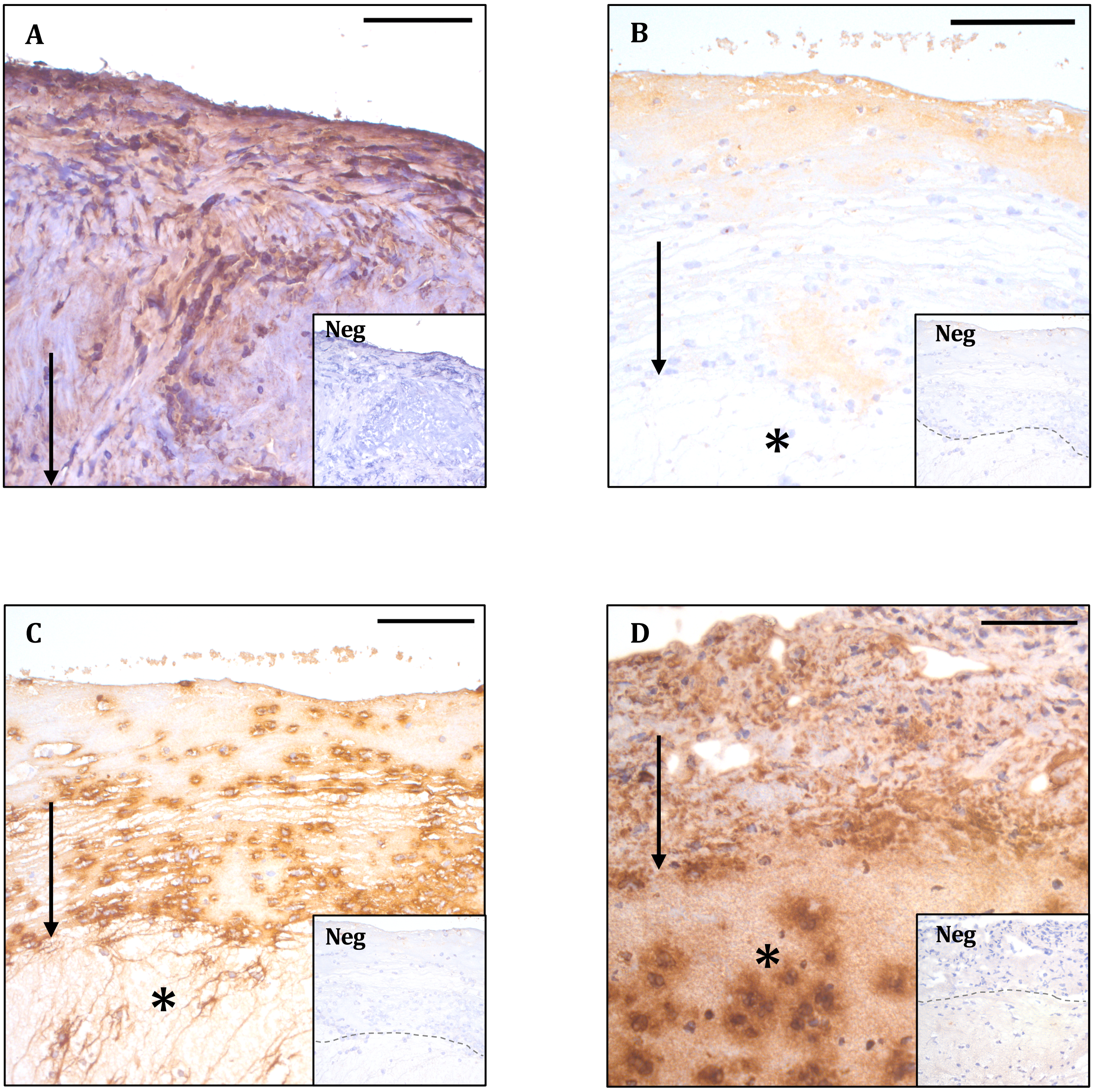

SUPPLEMENTAL FIGURE 1: Representative images of immunohistochemical stainings for matrix-metalloproteinase-2 (MMP-2) (panel A), cyclo-oxygenase 2 (COX2) (panel B), prostaglandin E 2 receptor (PGE2R) (panel C), and myeloperoxidase (MPO) (panel D) originating from thesame ruptured intracranial aneurysm as presented in panels C and E-H in Figure 1. Arrows point down towards the lumen. Thrombus is indicated with an asterisk. Negative controls are shown as insets. The interphases between the sIA wall and thrombus are indicated by dashed lines in the insets. Positive staining is indicated by brown. Hematoxylin background staining. Scale bars correspond to 100 μm.

Supplemental Figure 2

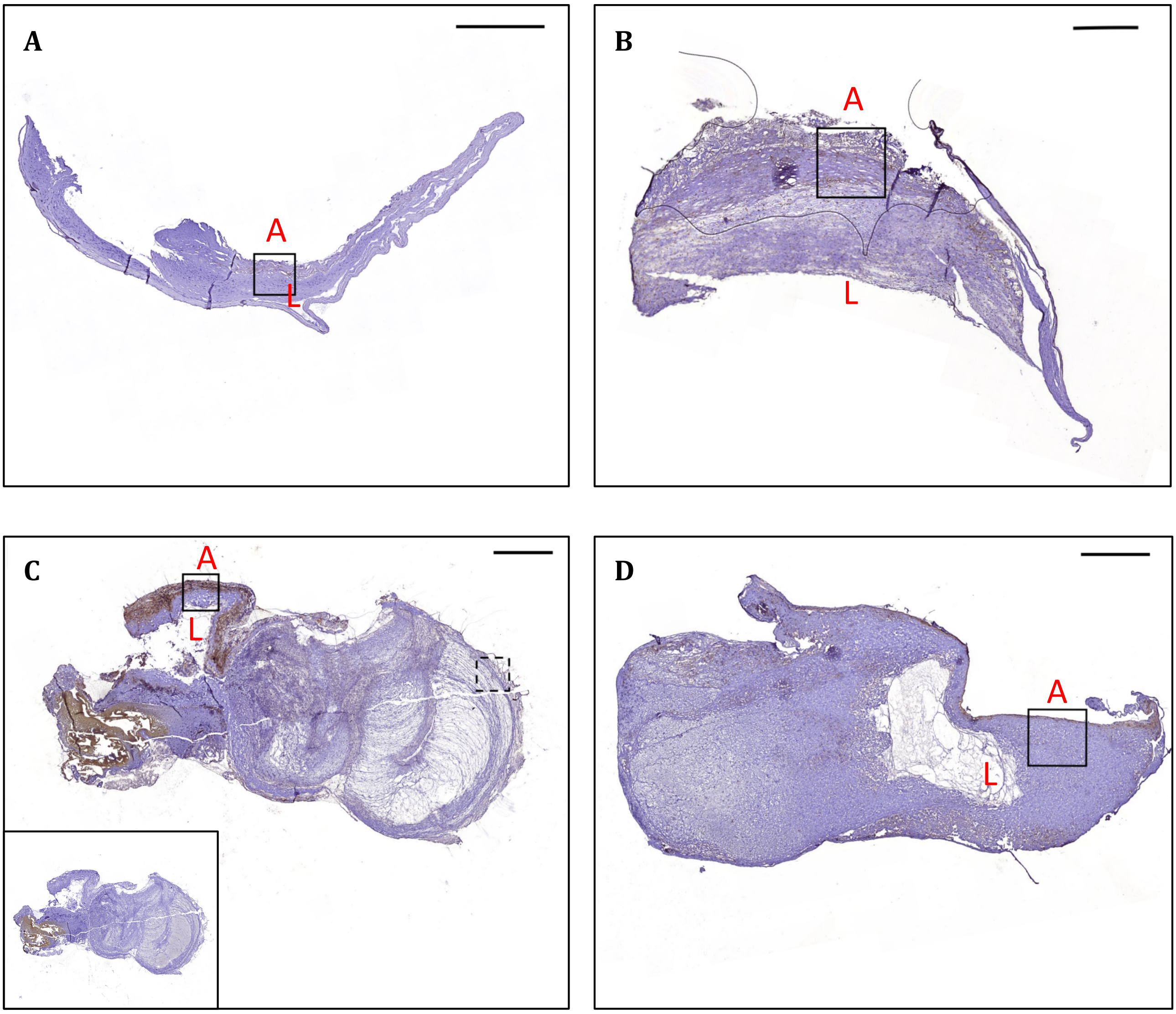

SUPPLEMENTARY FIGURE 2:
Representative low magnitude images of saccular intracranial aneurysm wall types A-D (panels A-D, respectively) of the same aneurysms as presented in Figure 1 (panels A-D, respectively). Small squares indicate the aneurysm wall areas presented in Figure 1 (panels A-D). In addition, the image in panel C is from the same as the aneurysm as presented in Figure 1 panels E-H and in Supplemental figure 1 panels A-D. Dashed square indicates the aneurysm wall area presented in Figure 1 panels E-H and in Supplemental figure panels A-D. Negative control for panel C is shown as an inset. Immunohistochemical staining for serum amyloid A. Scale bars correspond to 500 µm. A = adventitia, L = lumen.
